# Supplementary material for: A qualitative study exploring how patient engagement activities were sustained or adapted in Canadian healthcare organizations during the COVID-19 pandemic
Source: PLoS One. 2023 Mar 16;18(3):e0282890. doi: 10.1371/journal.pone.0282890 (PMC10019689; doi:10.1371/journal.pone.0282890)
Supplement: Attachment — Submitted filename: Response to reviewers_FINAL.docx [file pone.0282890.s001.docx]

January 27^th^, 2023

Dr. Sara Rubinelli

Academic Editor

PLOS ONE

**RE: Response to Reviews**

Submission ID: PONE-D-22-27751

Dear Dr. Rubinelli,

Thank you for seeing the potential in our manuscript and giving consideration for revisions that have strengthened our manuscript. We appreciate the reviewers’ thoughtful comments and have outlined a detailed response to each of the comments, along with a reference to the line numbers where the changes can be found in the main paper (i.e., revised manuscript with track changes).

| **Comment** | **Response** |
| --- | --- |
| **Reviewer #1** | |
| This paper is a quality job, and the results are very useful. I have a little to add about qualitative methodology. | Thank you for your comments and taking the time to review our manuscript. Your feedback has helped strengthen our manuscript. |
| It would be interesting for authors and readers if authors compare their conclusions with relevant quantitative studies and qualitative. In this way, they can assess and value if conclusions can be helpful for quantitative in the future.  Authors could argue more if these conclusions and comments of their work can help to create sustainable healthcare organisations in the future, and these can lead to more innovative procedures. I suggest you look at the following papers <https://doi.org/10.3390/su142215416>  <https://www.mdpi.com/2071-1050/12/7/2730> | Thank you for the suggestion. We have included the most recent literature when discussing our findings. However, there is little literature, to our knowledge, that is available due to the more recent nature of this topic. This is a strength that we have highlighted in our strengths and limitations section (lines 716-723)  Thank you for encouraging us to think about broader concepts and ideas from the literature. We feel that perhaps our findings support creating a more resilient health system, which could be done through knowledge management. Sharing of knowledge across organizations, including patient partner expertise and knowledge, is important for helping an organization withstand shocks and crises, while keeping patient partners engaged during a disruption. We have included a statement in the discussion to this point (lines 648-652). |
| **Reviewer #2** | |
| Dealing with the pandemic ad still involving the patients of different groups is a topic of great interest. The authors try to address this topic with a qualitative approach conducting qualitative study phases. This is interesting, even if only for a small target group. The manuscript is well-written but needs major revisions. For me there are a few questions that need to be addressed. | Thank you for your comments and taking the time to review our manuscript. Your feedback has helped strengthen our manuscript. We have addressed each of your questions below. |
| **Spacing and orthographies:** Throughout the manuscript there are various spacing issues. | Spacing has been addressed. |
| **Precision:** Throughout the manuscripts various paragraphs are very talkative and too long. More actual information would be beneficial. Please be more precise and give necessary information. | We have revised the overall manuscript to be more concise. |
| **Abstract** p.2, l. 32: What is defined as “organizations” in this case? | We have expanded our definition of organizations to include healthcare, government and patient partner organizations (at a policy and practice level of healthcare) in the abstract as well as througout the manuscript. |
| **Introduction:** The manuscript lacks some links between the introduction section and its theories with the final results of the current study. This should be more precise. | We have strengthened the introduction to better link with the final results of the study. |
| Throughout the sections “introduction”, “methods” and “results” there are several occasions where authors use personal statements (we, our, etc) This should be deleted. Personal statements and comments should only be included in the discussion section. | Personal statements have been removed throughout the paper. |
| **Materials and Methods:** p. 4, ll.83: This has been said a couple of time before. The authors should focus on more precision and avoid giving certain statements at different pages of the manuscript again and again. | The repetition has been removed in the introduction and methods section. |
| p. 5, ll.94: “various healthcare organization”. What is meant by this? What define the authors as healthcare organization? So far, the reader have no idea on where the interviews were conducted. It’s a big difference if local support groups or a university hospital manager was interviewed. | In the abstract and introduction, we have identified that we are referring to healthcare, government and patient partner organizations that operate at a policy or practice level of healthcare. We now only refer to these as organizations throughout the paper for simplicity. |
| p. 5, ll.94: Why informal discussions? That is not scientifically sound. Was an interview guide used? Where the discussions transcribed? | The information obtained from these discussions was not part of the data collection but rather used to inform the selection of cases/recruitment. The use of informal conversations has been applied in qualitative research (see ref below). We have identified the purpose of these conversations earlier in the environmental scan section so it is more clear to the reader how these conversations informed our study (line 121).  Reference: Swain, J., & King, B. (2022). Using Informal Conversations in Qualitative Research. International Journal of Qualitative Methods, 21. https://doi.org/10.1177/16094069221085056 |
| p. 6, l. 109: “eight different organizations”. Please clarify the process. Which organizations? | The process of choosing the eight organizations is described in the environmental scan section (lines 121-133).  In case selection and recruitment, we have described the 8 organizations chosen (lines 141-145) |
| p. 6, l.114ff: Concerning the eligibility criteria: What about consenting? Was consent obtained? Where participants included without consent? | Consent is discussed in the data collection section as part of the data collection process (lines 161-165). All participants who were interviewed provided consent to be part of the study. There were no participants included without consent. |
| p. 6, l. 125: verbal consent: See comment above. A verbal consent is useless. Respondents need to give written consent in order to use the data. | As per our research ethics board, we were approved to obtain either written or verbal consent. This was due to the online nature of the interviews as a result of the pandemic and to increase accessibility for participation (e.g., some participants do not own a printer or scanner to sign and send back consent forms). We have added a line to indicate that consent was documented by the researcher who obtained consent in both the consent form and the recruitment log and kept separate from the interview (line 163-165). |
| p. 7, l. 146ff: There seems to be a problem with formatting. The color is changing. | We have ensured all text is in black text. |
| **Results:** In general: This section is rather challenging and needs more explanation | We have reviewed our results and have done the following to increase the clarity of our findings: removed any redundancy, removed selected quotes, and provided summary statements and subheadings in each section (also summarized in a table). |
| p. 8, l. 165: For this study in general: The manuscript would highly benefit from a clear definition what authors mean by “partnership” and “patient activity”. This should be added | A definition is provided in the introduction (lines 67-71:  “Beyond being engaged in their direct care, patients and their caregivers/families can collaborate and partner with researchers and health professionals across all levels of the healthcare system in a range of patient engagement activities, such as planning, research, evaluation, consultation, and decision making [9, 10].” |
| The whole section is way too long and uses way too many quotes. There are no actual results presented. | We have revised the results to be more concise, including the removal of some quotes. We have presented our main findings as 4 key themes while using quotes as our empirical evidence, as per the Standards for Reporting Qualitative Research (SRQR). We have also included sub-themes to help the flow of the results (see Table 1)  Reference: O’Brien BC, Harris IB, Beckman TJ, Reed DA, Cook DA. Standards for reporting qualitative research: a synthesis of recommendations. Academic medicine. 2014;89(9):1245-51. |
| **Discussion:** In general, the discussion of the results can be shortened. There are several paragraphs that just repeat statements given I the results section already. The authors should be more precise and discuss the impact of there findings. What is the take home message? What can other hospitals learn from the examples presented. | We have revised the discussion to be more precise and have revised our conclusion to have clear take home messages for other organizations to learn from our findings. |
| p.25, l.537: This is already mentioned on page 20, lines 412ff..should be deleted here | This section has been revised to be more concise and less repetitive. |
| p. 26, l. 552-554: This has been stated several times before. Please be more precise and avoid repetitions. | We have reworded the opening sentences to highlight our study strengths while being less repetitive (lines 713-720) |
| **Conclusion:** p. 27, l. 575: please see comment on p. 26, l. 552-554. This is said again and again. - | We have revised throughout the manuscript to limit the repetition of similar statements. |
| The conclusion is too vague and too generally valid and not specifically adjusted to the study. More details on how the study finding add value to the ongoing discussion should be given.  At the beginning of the manuscript a multi-center study is mentioned. How do the results of this study part influence to overall study? | We have revised the conclusion to include clear take home messages, the impact of the work and what organizations can learn from our findings.  This paper is focused on an analysis that was part of a larger qualitative, case study. We have revised the methods section (lines 116-118) to be clear that this was not a separate study. |
| **References:** Reference 33: Why is the PMID given? | This has been removed. |
